# The Role of Web-Based Health Information in Help-Seeking Behavior Prior to a Diagnosis of Lung Cancer: A Mixed-Methods Study

**DOI:** 10.2196/jmir.6336

**Published:** 2017-06-08

**Authors:** Julia Mueller, Caroline Jay, Simon Harper, Chris Todd

**Affiliations:** ^1^ School of Health Sciences University of Manchester Manchester United Kingdom; ^2^ Manchester Academic Health Science Centre Manchester United Kingdom; ^3^ School of Computer Science University of Manchester Manchester United Kingdom

**Keywords:** help seeking, online health information, health information seeking, lung cancer, symptom appraisal

## Abstract

**Background:**

Delays to diagnosis in lung cancer can lead to reduced chance of survival, and patients often wait for several months before presenting symptoms. The time between first symptom recognition until diagnosis has been theorized into three intervals: symptom appraisal, help-seeking, and diagnostic interval (here: “pathway to diagnosis”). Interventions are needed to reduce delays to diagnosis in lung cancer. The Web has become an important lay health information source and could potentially play a role in this pathway to diagnosis.

**Objective:**

Our overall aim was to gain a preliminary insight into whether Web-based information plays a role in the pathway to diagnosis in lung cancer in order to assess whether it may be possible to leverage this information source to reduce delays to diagnosis.

**Methods:**

Patients diagnosed with lung cancer in the 6 months before study entry completed a survey about whether (and how, if yes) they had used the Web to appraise their condition prior to diagnosis. Based on survey responses, we purposively sampled patients and their next-of-kin for semistructured interviews (24 interviews; 33 participants). Interview data were analyzed qualitatively using Framework Analysis in the context of the pathway to diagnosis model.

**Results:**

A total of 113 patients completed the survey (age: mean 67.0, SD 8.8 years). In all, 20.4% (23/113) reported they or next-of-kin had researched their condition online before the diagnosis. The majority of searches (20/23, 87.0%) were conducted by or with the help of next-of-kin. Interview results suggest that patients and next-of-kin perceived an impact of the information found online on all three intervals in the time to diagnosis. In the appraisal interval, participants used online information to evaluate symptoms and possible causes. In the help-seeking interval, the Web was used to inform the decision of whether to present to health services. In the diagnostic interval, it was used to evaluate health care professionals’ advice, to support requests for further investigation of symptoms, and to understand medical jargon. Within this interval, we identified two distinct subintervals (before/after relevant diagnostic tests were initiated), in which the Web reportedly played different roles.

**Conclusions:**

Because only 20.4% of the sample reported prediagnosis Web searches, it seems the role of the Web before diagnosis of lung cancer is at present still limited, but this proportion is likely to increase in the future, when barriers such as unfamiliarity with technology and unwillingness to be informed about one’s own health are likely to decrease. Participants’ perceptions suggest that the Web can have an impact on all three intervals in the pathway to diagnosis. Thus, the Web may hold the potential to reduce delays in the diagnostic process, and this should be explored in future research and interventions. Our results also suggest a division of the diagnostic interval into two subintervals may be useful.

## Introduction

Lung cancer is the leading cause of cancer deaths worldwide [1]. Low survival rates for lung cancer have been linked to delays to diagnosis [2]; the majority of patients are diagnosed at advanced disease stages, which decreases chance of survival [3].

**Figure 1:**
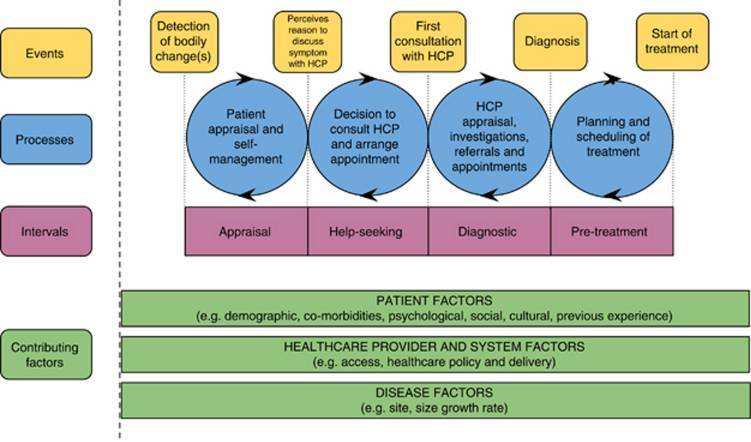
Model of pathways to treatment (from Walter et al [4]).

The route from symptom recognition to diagnosis and commencement of treatment has been theorized into four intervals by Walter et al [4] in a model of pathways to treatment (Figure 1). In the first interval, the “appraisal interval,” an individual appraises and interprets bodily changes. This is followed by the “help-seeking interval,” in which the individual decides whether to consult a health care professional about the bodily changes [5]. The following “diagnostic interval” involves appraisal by health care professionals, investigations, referrals, and appointments. In the event of a diagnosis, the “pretreatment interval” then commences, which involves planning and scheduling of treatment. The length of these intervals can be influenced by disease factors (eg, site, size, growth rate), health care provider and system factors (eg, access to resources, health care policy), and patient factors (eg, psychosocial factors).

Here we focus on the three intervals leading up to diagnosis (appraisal, help-seeking, and diagnostic; Figure 1) because low survival rates in lung cancer have been linked to delays to diagnosis [2,6]. We refer to these three intervals as the “pathway to diagnosis.”

We focus on patient factors because research has shown that people with lung cancer often experience symptoms for several months before presenting to health services [7-9]. Research suggests lack of knowledge about lung cancer symptoms is one of the biggest barriers to help-seeking [7,9-14]. Furthermore, symptoms are often masked by preexisting comorbidities that have similar symptoms, making it difficult for the patient to distinguish between existing and new symptoms [12,15]. Fear of being diagnosed with cancer and fatalistic beliefs about treatability of lung cancer may also impede help seeking [8,12].

Lung and colorectal cancer patients have been shown to be proactive when appraising symptoms [14] and health-related Web use has been documented in various cancer populations, such as lung, colorectal, prostate, testicular, breast, cervical, and bowel cancer [16-18]. This suggests the Web could play a role in the time before cancer diagnosis (eg, if people with cancer search the Web for information to appraise their symptoms). Although evidence indicates that people with lung cancer do access the Web [18,19], the proportion of Web users is likely to be low because lung cancer patients tend to be older (>70 years) and to have lower education levels and socioeconomic status [1], and these factors are related to low levels of health-related Web use [20]. Overall, there is a growth of health information on the Web and an increasing tendency for individuals to seek health information online [21]. However, little is known about how people make use of this source prior to diagnosis because most research focuses on Web usage after patients have been diagnosed.

Due to the scarcity of previous research on this topic, an exploratory approach was required to gain a preliminary understanding of the potential role of online health information during the time leading up to a lung cancer diagnosis. In this study, we aimed to gain this preliminary understanding by exploring patients’ own retrospective accounts of how they remember the events leading up to their diagnosis, with particular focus on the perceived impact of Web searches on this process. Previous research has shown that family members sometimes conduct Web searches on behalf of patients [17] and that family members play an important role in lung cancer patients’ help-seeking behavior [7,8,11,22]. Therefore, we also aimed to explore accounts of next-of-kin of patients and whether they assisted patients with online searches or conducted searches on their behalf.

Our overall aim was to gain a preliminary, exploratory insight into whether Web-based information plays a role in the pathway to lung cancer diagnosis. This is important because, if the Web is found to play a role, it may be possible in the future to leverage this information source to reduce delays to diagnosis. To meet this aim, we addressed three research questions:

1. What proportion of people with lung cancer (or their family/friends) retrospectively report researching their condition online prior to diagnosis?
2. In cases in which prediagnosis Web searches take place, how do individuals perceive the impact of the information they find on their pathway to diagnosis?
3. What are possible barriers to using the Web prediagnosis?

## Methods

### Design

Our research questions required the combination of quantitative and qualitative methods. Quantitative methods were used to establish the proportion of lung cancer cases in which prediagnosis Web searches took place (question 1). Qualitative methods were used to explore individuals’ perceptions of the impact their Web searches had on the pathway to diagnosis, as well as barriers that might prevent individuals from accessing the Web for health information prediagnosis (questions 2 and 3). Finally, mixed methods were required because a survey was needed to screen for relevant individuals for interview because we expected low levels of Web use among lung cancer patients.

Thus, this study consisted of (1) a cross-sectional, retrospective survey and (2) a qualitative interview study with a subsample of the survey participants.

### Participants and Recruitment

We recruited recently diagnosed lung cancer patients to explore patients’ retrospective accounts of the events leading up to their diagnosis. Participants were recruited from outpatient clinics at two large university hospitals in the northwest of England between July 2014 and March 2015. Patients were eligible if they (1) had received a lung cancer diagnosis in the 6 months prior to study entry, (2) had sufficient English language to complete the questionnaire, (3) were able to consent, and (4) reported experiencing at least one symptom before diagnosis. Patients whose diagnosis was more than 6 months before study entry were excluded to reduce recall bias [17].

Participants were sampled for interviews purposively based on questionnaire responses regarding (1) whether the Web had been used prior to diagnosis and (2) sociodemography (age, gender, smoking status) because these sociodemographic factors have been shown to be related to Web use [23]. We included both Web users and non-Web users to gain insight into reasons for and against using the Web prior to diagnosis. Next-of-kin were invited to participate in interviews because they tend to be involved in health information seeking [24] and have been shown to play an important role in lung cancer patients’ help seeking [7,8,11,22]. We recruited next-of-kin who engaged in Web searches prior to diagnosis on the patient’s behalf and those who did not. Data collection continued until saturation was reached (ie, when no new evidence for theoretical points emerged and we began to note similar accounts recurring) [25].

### Procedure

We approached patients attending outpatient clinics, who had previously been identified from clinical notes as potentially eligible. Following informed consent, we provided participants with a questionnaire and stamped-addressed return envelope, with the option of completing the questionnaire in clinic (with the researchers, if they wished) or at home. A subset of consenting participants was selected purposively to participate in follow-up interviews, which were conducted in clinic.

### Measures

#### Questionnaire

The paper-based questionnaire took 10 to 15 minutes to complete. Questions were standardized and assessed (1) whether the patient and/or a family member/friend had used the Web prior to diagnosis to help understand the symptoms/condition, (2) which symptoms were experienced before the diagnosis, (3) details on Web searches conducted prior to diagnosis if applicable (who conducted the search, search engine and search terms used, websites accessed), (4) information on habitual Web/technology use (whether the Internet is ever used; if yes, number of hours during a typical week), and (5) sociodemographic information (age, sex, education level, and employment status).

The development of the questionnaire was informed by previous literature on help-seeking behavior and Web-searching behavior [18,23,26], medical reference works [27,28], discussion with a Patient and Public Involvement group for cancer and palliative care, as well as brainstorming within the research group.

#### Interviews

Interviews were semistructured with open-ended questions and standardized prompts. The interview topic guide covered:

1. Symptom experience prior to diagnosis, with a focus on motivators and barriers to seeking help;
2. Web searches conducted prior to diagnosis (if applicable), with a focus on perceived impacts on the pathway to diagnosis (eg, the decision of whether to present to health services); and
3. Reasons for and against using the Web prior to diagnosis.

#### Clinical Records

Following consent, type of lung cancer and smoking status were obtained from patient records.

### Analysis

#### Quantitative Analysis

We analyzed questionnaire data descriptively using IBM SPSS version 22 to calculate percentages, means, and standard deviations. For proportions, we calculated 95% confidence intervals using Confidence Interval Analysis (CIA) version 2.2.0 [29] as an indication of the variability of the results and to facilitate comparisons. Group differences in continuous variables were tested using the nonparametric Mann-Whitney *U* test, and associations between categorical variables were tested using Fisher's exact test, with Cramer's *V* computed to assess effect size.

#### Qualitative Analysis

Interviews were audio-recorded, transcribed verbatim, and organized using QSR NVivo10. Framework Analysis [25] was used to identify recurring and important themes in the data. Our analysis involved the following five stages [30,31]:

##### Familiarization With the Data

Familiarization was achieved by repeatedly reading all interview transcripts and noting recurring topics.

##### Development of a Theoretical Framework

A broad framework of topics was developed to organize the data, based on the interview protocol as well as recurring topics identified in step 1. The topics were then sorted and grouped under broad categories to create a hierarchical structure of topics and subtopics.

##### Indexing Data

The framework was then applied to the data by using NVivo to label transcript sections according to the topics occurring in each section. This was undertaken by at least two independent researchers and any discrepancies discussed until consensus was reached.

##### Summarizing Data in Thematic Charts

A matrix was created within NVivo10 [32] for each topic, with participants in the rows and subtopics in the columns. Transcript sections were then summarized into the relevant cells, keeping as close to participants’ original wording as possible. To illustrate, an excerpt from a framework matrix is provided in Multimedia Appendix 1.

##### Synthesizing Data by Mapping and Interpreting

Matrices were next explored by comparing cells across participants and within participants to identify similarities or differences in how participants described their experiences. This facilitated identification of recurring themes and links between themes. Themes were discussed in the research group until consensus was reached. To aid interpretation, we categorized participants’ reported Web searches according to the interval in the pathway to diagnosis [4] in which they occurred. Searches were assigned to the appraisal interval if they took place before the searcher perceived a reason to present the symptoms to a health care professional. They were classed as help-seeking interval if they took place between perceiving a reason to present symptoms and first consultation. Searches were assigned to the diagnostic interval if they took place after first consultation, but before a diagnosis was given.

## Results

### Survey

#### Sample Description

**Figure 2:**
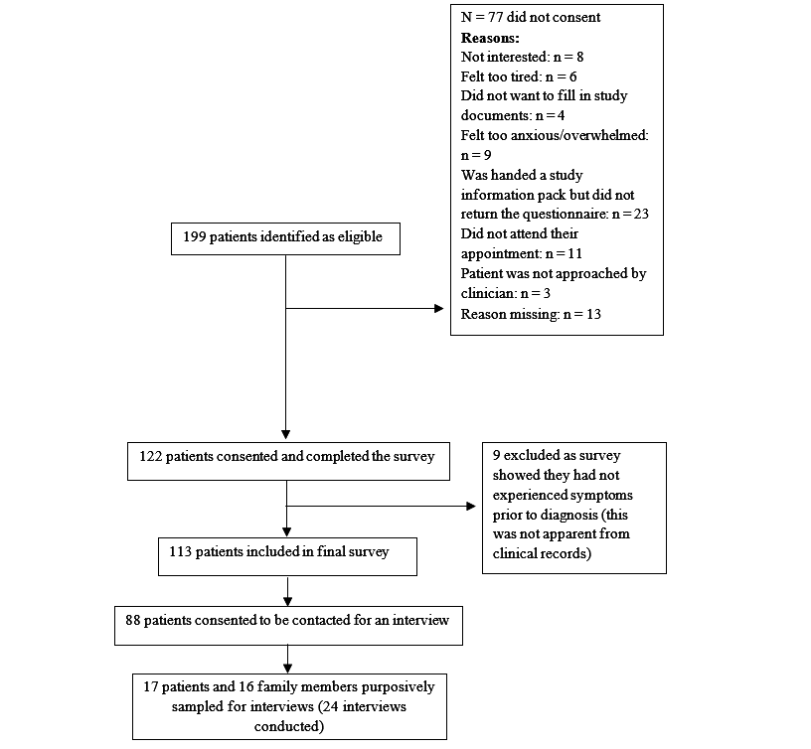
Participant recruitment flow diagram (STROBE diagram).

Between July 2014 and March 2015, 199 patients were identified as eligible and 122 consented (61.3%). Nine participants were excluded after consent because they had not experienced symptoms prior to diagnosis (it was not possible to discern this in advance from clinical records); therefore, 113 participants were included in the final sample (Figure 2). The mean age was 67.0 (SD 8.8) years and ranged from 42 to 88 years. The majority were male (56.6%, 64/113), retired (65.5%, 74/113), former smokers (69.0%, 78/113), and reported educational attainment below university level (82.3%, 93/113); 48.7% (55/113) had non-small cell lung cancer (Table 1).

Approximately half of all participants had an Internet connection (51.3%, 58/113) and 61.1% (69/113) had used an Internet device at some point; 23.0% (26/113) did not own any Internet device (Table 2).

**Table 1:** Participant demographic details (N=113).

Sociodemographic variables		Parameters
**Age (years), mean (SD)**		
	Male	66.7 (8.7)
	Female	67.1 (8.9)
	Total	67.0 (8.8)
**Gender, n (%)**		
	Male	64 (56.6)
	Female	49 (43.4)
**Employment status, n (%)**		
	Employed	24 (21.2)
	Unemployed	12 (10.6)
	Retired	74 (65.5)
	Missing	3 (2.7)
**Education level, n (%)**		
	No formal education	16 (14.2)
	Secondary or high school	19 (16.8)
	GCSE or equivalent	22 (19.5)
	A levels or equivalent	4 (3.5)
	Vocational qualification	22 (19.5)
	Professional qualification	10 (8.8)
	University degree	6 (5.3)
	Missing	14 (12.4)
**Type of lung cancer, n (%)**		
	Non-small cell lung cancer	55 (48.7)
	Small cell lung cancer	44 (38.9)
	Combined	1 (0.9)
	Missing	13 (11.5)
**Smoking status, n (%)**		
	Never-smoker	3 (2.7)
	Former smoker	78 (69.0)
	Current smoker	25 (22.1)
	Missing	7 (6.2)

**Table 2:** Habitual Web/technology use among participants (N=113).

Item	n (%)
Internet connection at home	58 (51.3)
Use of a computer	60 (53.1)
Owning a computer	73 (64.6)
Use of a Web-enabled mobile phone	24 (21.2)
Owning a Web-enabled mobile phone	40 (35.4)
Use of a tablet	34 (30.1)
Owning a tablet	38 (33.6)
Ever use any Internet devices	69 (61.1)
Not owning any Internet devices	26 (23.0)

**Figure 3:**
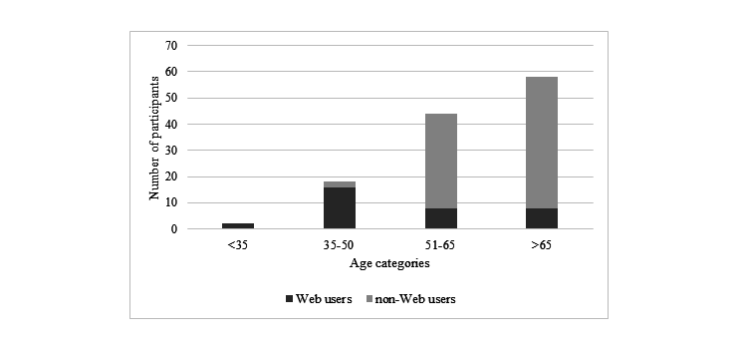
Age distribution of Web users (those who used the Web prior to diagnosis to help them understand symptoms) and non-Web users.

#### Proportion of People With Lung Cancer (or Their Family/Friends) Who Reported Web Searches Prior to Diagnosis

Of the sample, 20.4% (23/113, 95% CI 12.9%-27.8%) reported they, or a family member/friend, researched symptoms online prior to diagnosis. Seven of 113 (6.2%, 95% CI 1.8%-10.6%) stated they researched their symptoms themselves, four of these with the help of a family member/friend. Family/friends conducted online searches on the patient’s behalf in another 16 cases (14.2%, 95% CI 7.7%-20.6%), thus the majority of searches (20/23, 87.0%) involved family/friends. Although 23 people reported the Web was used in their case, some reported several Web users (eg, a spouse and grandchild). Thus, in total, we identified 31 Web users: 7 patients, 7 spouses/partners, 12 sons/daughters, 2 sons-in-law/daughters-in-law, 1 grandchild, 1 nephew, and 1 friend. The age distribution of Web users is shown in Figure 3.

#### Description of Web Searches

Of the 23 participants who reported Web searches prior to diagnosis, 20 reported that Google was used to search; the rest did not know which search engine was used. The majority (19/23) reported using the NHS Direct website. Other websites included WebMD (7/23), patient.co.uk (5/23), Yahoo Health (2/23), and Netdoctor (1/23). Two participants reported visiting discussion forums.

Twenty-one participants reported search terms used (Table 3). Eight participants used symptoms as search terms (eg, “persistent cough”) and five used possible causes/conditions, such as “throat cancer” or “stopping smoking.” Three people used investigative test results that had been communicated to them by health professionals before they had received a final diagnosis (eg, “pleural effusion”). In these three cases, the Web searches took place before a diagnosis was given, but after some investigation of symptoms had been initiated. Five participants used combinations of these (eg, “stomach cancer and weight loss”). Overall, five participants reported that “lung cancer” was included in their search.

#### Web Searches and Symptoms

The majority (67.3%, 76/113) of participants reported experiencing a cough prior to diagnosis (Table 4). On average, participants reported mean 3.0 (SD 1.6) symptoms.

**Table 3:** Search terms used during Web searches prior to diagnosis (n=23).

Category and terms		Participants, n
**Symptoms**		
	Persistent cough	4
	Hoarse voice, hoarseness, croaky voice	3
	Back pain, shoulder pain, lower back pain	2
	Coughing up blood	1
	Lump on neck	1
	Dyspnea	1
	Swollen face and neck	1
	Recurrent chest infection	1
	Pain in chest	1
	Weight loss	1
	Night sweats	1
	Dry mouth	1
**Conditions / possible causes**		
	Lung cancer, lung cancer symptoms	5
	Cancer	1
	Throat cancer	1
	Stomach cancer	1
	Myasthenia	1
	Stopping smoking	1
	Anxiety	1
**Test results (communicated to the patient before the final diagnosis was given)**		
	Pleural effusion, fluid in lungs	1
	Patch on vocal cords	1
	Iron anemia	1
	Collapsed lung	1

**Table 4:** Number and percentage of participants reporting respective symptoms (N=113).

Symptom	n (%)
Cough	76 (67.3)
Change in an existing cough	22 (19.5)
Hemoptysis	19 (16.8)
Dyspnea	59 (52.2)
Fatigue	51 (45.1)
Weight loss / loss of appetite	44 (38.9)
Shoulder/back pain	15 (13.3)
Chest pain	13 (11.5)

**Table 5:** Association between reporting symptoms and reporting Web use prior to diagnosis.

Reported symptoms		Web was used, n (expected count)	Web was not used, n (expected count)	χ^2^_1_	*P* (Fisher's exact test)	Cramer's *V*
**Cough**				0.7	.46	0.076
	Yes	14 (15.6)	62 (60.4)			
	No	9 (7.4)	27 (28.6)			
**Change in an existing cough**				0.1	>.99	0.074
	Yes	3 (4.3)	18 (16.7)			
	No	20 (18.7)	71 (72.3)			
**Hemoptysis**				0.3	.76	0.053
	Yes	3 (3.9)	16 (15.1)			
	No	20 (19.1)	73 (73.9)			
**Dyspnea**				0.7	.49	0.079
	Yes	14 (12.2)	45 (46.8)			
	No	9 (10.8)	43 (41.2)			
**Fatigue**				0.5	.64	0.065
	Yes	9 (10.5)	42 (40.5)			
	No	14 (12.5)	47 (48.5)			
**Weight loss**				3.6	.09	0.179
	Yes	13 (9.0)	31 (35.0)			
	No	10 (14.0)	58 (54.0)			
**Shoulder/back pain**				<0.1	>.99	0.005
	Yes	3 (3.1)	12 (11.9)			
	No	20 (19.9)	77 (77.1)			
**Chest pain**				0.2	>.99	0.046
	Yes	2 (2.7)	11 (10.3)			
	No	21 (20.3)	78 (78.7)			

The number of symptoms reported by the patient was not significantly associated with whether the Web was used prior to diagnosis (*U*=1041, *Z=* –0.19, *P=*.85). As Table 5 shows, having any particular symptom was not significantly associated with whether the Web was used prior to diagnosis.

### Qualitative Interviews

Twenty-four interviews were conducted: 10 with patients, 7 with next-of-kin, and 7 with the patient and next-of-kin together. In total, 33 people were interviewed (n=19 female). Fourteen interviews involved Web searches prior to diagnosis, either by the patient or next-of-kin.

#### Perceptions of the Impact of Prediagnosis Web Searches on the Pathway to Diagnosis

In the following, we have grouped Web searches according to the time intervals in Walter et al’s model [4] during which they occurred. Within each interval, we explore participants’ perceptions of how their Web searches impacted the processes described in the model.

##### Appraisal Interval: Perceived Impacts on Appraisal and Self-Management

Some participants reportedly accessed the Web for information once they had perceived bodily changes, but had not yet decided to present these to a health care professional. Online information was used to identify possible causes of symptoms:

And [husband’s name] being how he is, he won’t go to the doctors anyway, so we did sort of self-diagnose, if you like.

R27, wife of patient, 51-65 years

Some participants described that the information they read online about symptoms changed the way they appraised their symptoms, causing them to view symptoms as more serious than before and, in some cases, even convincing them that the cause was lung cancer:

I just put in, to start with, shoulder pain, and lung cancer came up straight away...And that’s...I thought, oh, you know; I looked at it, and I thought, lung cancer? Crikey! Because I’d no idea that people got pain anywhere near there. And so I went on one website after another, after another, after another, just to try and read the symptoms to see if the symptoms were all the same on each site, and they were, basically.

R18, daughter of patient, 51-65 years

One participant described that the information she found online caused her to view her husband’s symptoms as less serious. In this particular case, the searcher (a patient’s spouse) entered the symptoms experienced by her husband as search terms, coupled with her hypothesis of what was causing the symptoms (ie, smoking cessation). For example, she reportedly searched for “stopping smoking and cough.” Using this search strategy, she reportedly felt reassured by the information she found that the symptoms were caused by smoking cessation rather than a disease:

R27: “You see, the sweating all night and the coughing. We’d had a look online and his friends had told him, he’d stopped smoking. So that happens, you get insomnia, you can’t sleep either and you’re coughing a lot. And you’re just bringing anything up that’s been in your lungs for years. So we sort of left that at that, thinking that’s what it was...”

Interviewer: “And in your search, did you, at any point, come across any information about lung cancer?”

R27: “I don’t think I did...my specific search terms were, stopping smoking, so I kept putting stuff with smoking in. I didn’t put night sweats, if you like, and get the whole...the amount of what would cause it, if you like.”

[...]

Interviewer: “Could you just tell me a little bit more about how you went about your search?”

R27: “So I probably put, stopping smoking and night sweats. And then what’s come up about that. Stopping smoking and cough...how long after stopping smoking, will they carry on coughing?”

R27, wife of patient, 51-65 years

#### Help-Seeking Interval: Perceived Impact on the Decision to Consult a Health Care Professional

According to Walter et al’s model [4], individuals in the help-seeking interval form the decision to consult a health care professional and make an appointment, and the interval is concluded when a first consultation takes place. Several participants in our interview study reportedly used online information to inform their decision on whether to present to a health care professional:

I kept thinking, this cough’s not clearing. But like I said, it went on months and months...so that’s when my son went on the Internet, and that’s when he said, “Mum, Aunt [name] and Aunt [name] they’re right, you need to go.” And that’s when I went.

R22, patient, >65 years

Some participants who conducted Web searches before first consultation with a health care professional perceived no impact on their decision making because they had reportedly already formed the decision to present to health services before they began their search:

I knew there was something wrong, that you had to go and see a doctor...The decision was made before I even googled it, yes.

R4, patient, 51-65 years

#### Diagnostic Interval: Perceived Impact on Health Care Professionals’ Appraisals

In Walter et al’s model [4], the diagnostic interval commences following first consultation and involves appraisal by a health care professional, investigations, referrals, and appointments. Within this interval, we identified two distinct subintervals at which Web searches took place: (1) after a health care professional had been consulted, but before relevant diagnostic tests (chest x-ray, CT scan) had been conducted, and (2) after relevant diagnostic tests had been conducted, but before a diagnosis had been communicated. Within these subintervals, Web searches had different perceived impacts.

##### Before Diagnostic Tests Were Underway

Participants who reported Web searches in this subinterval described presenting to health services multiple times without diagnostic tests to determine the cause of the symptoms. Participants reportedly turned to the Web because they felt dissatisfied with the advice they received from health care professionals:

So I went on the Internet, I think because she’d had four visits to the doctor and we weren’t getting anywhere, so I went on to just see, you know, if I could find anything out really to give me an idea what else it could be other than an allergic reaction.

R14, daughter of patient, 51-65 years

Interviewees reportedly used the information found online to challenge their doctors’ advice by suggesting other possible causes for symptoms and requesting further tests. Participants felt that their assertiveness in urging further investigation impacted on health care professionals’ decisions to conduct diagnostic procedures:

So then I went online and I put in facial swelling and neck swelling and it said it could be an infection of the glands or the ducts. So that’s why I asked the GP, when I went back with her on the fifth visit, could it be an infection in the glands or the ducts...I’m not saying he wouldn’t have done it but I think the fact that I was with my mum and maybe being a little bit more assertive instigated him to maybe look a little bit further. Yeah, definitely...I wasn’t rude but I was assertive, and it was only then that he investigated further and listened more closely to her chest.

R14, daughter of patient, 51-65 years

##### After Diagnostic Tests Were Underway

Participants often described a period of several weeks during which diagnostic tests were undertaken and results communicated to them, but they were not informed of what these test results could mean. During this period of uncertainty, participants reportedly conducted online searches to understand medical jargon and test results, and to identify possible causes:

He said, it looks like one of the lungs have collapsed, but obviously we need to go and see a specialist at the [university hospital], which is what we did. But prior to actually seeing a specialist, I started looking then, on what could cause a collapsed lung.

R27, wife of patient, 51-65 years

#### Perceived Barriers to Using the Web Prediagnosis

We also interviewed patients and next-of-kin of patients who had not accessed the Web prior to diagnosis in order to understand perceived barriers to prediagnosis Web use. This can help to understand whether the role of the Web in the pathway to lung cancer diagnosis may change in the future and if barriers are aspects that are likely to change or not.

##### Concern Over Unnecessary Worry and Fear

Of those who reported not researching their condition online prior to diagnosis, several reportedly avoided this because they were concerned that it could lead to unnecessary worry and fear:

Sometimes it can frighten the life out of you, you know what I mean? It’s like when people used to buy the home medical directory and you’d got a headache and something else, when you looked it up, you’ve got everything under the sun.

R8, husband of patient, >65 years

##### Preferring Not to Know

Some participants stated that they wanted to know as little as possible about their health, preferring to leave decisions to health professionals:

No I don’t like looking it up. I don’t really like knowing unless I’ve got to.

R21, patient, 51-65 years

##### Believing Symptoms Trivial

One participant felt her symptoms were too mild and familiar to warrant further research:

I mean I like to know what’s going on but I wouldn’t research a tickly cough because I’ve been in that situation many times before so...

R6 patient, patient, >65 years

##### Unfamiliar With or Not Interested in Technology

Most of those who did not research their condition online were not comfortable using technology:

Interviewer: “Do you ever use the computer?”

R24: “No, because I can’t even, it takes me all this time to text and reply. I prefer a conversation, you know, ring somebody. You know, further than that, I just make a mess of everything.”

R24, patient, >65 years

Some expressed disinterest in the use of technology:

I am computer illiterate...and I prefer it that way. Yeah. I used to use a computer when I worked, it’s not that I can’t, it’s that I’m not interested; it’s such a waste of time.

R6, patient, 51-65

A few participants wanted to access the Internet, but lacked the skill:

The lads have got it now and I think they, I think they are brilliant. I wish it’s one of the things, I wish I could, but I’ve never gone onto the Internet.

R21, patient, 51-65 years

## Discussion

This is the first study to explore prediagnosis Web searches among lung cancer patients. We found that approximately a fifth of the sample of lung cancer patients reported prediagnosis Web searches to research symptoms and help them understand their condition, with most searches conducted by next-of-kin. Furthermore, our analyses showed that patients and their next-of-kin perceived impacts of their prediagnosis Web searches on their pathways to diagnosis, including symptom appraisal, forming the decision to seek help, and interactions with health care professionals.

Our overall aim was to gain a preliminary, exploratory insight into whether Web-based information plays a role in the pathway to lung cancer diagnosis. To explore this role, we discuss subsequently (1) the proportion of people with lung cancer reporting prediagnosis Web searches, (2) perceived impacts of the Web searches on the pathway to diagnosis, and (3) what prevents people from accessing the Web and whether this is likely to change in future.

### Proportion of People With Lung Cancer Reporting Prediagnosis Web Searches

We found that 20.4% (23/113) of our sample reported Web searches prior to diagnosis to help appraise symptoms or understand their condition. The majority of searches were conducted by or with the help of a family member. Although more than half (61.1%, 69/113) of the patients in the survey indicated having used the Internet in the past, and approximately half (51.3%, 58/113) reported having an Internet connection at home, only 6.2% (7/113, 95% CI 1.8%-10.6%) of patients reported researching their condition online themselves.

Only one other published study has examined the proportion of cancer patients who engage in prediagnosis Web searches. In a study with colorectal cancer patients, Thomson et al [17] found 25% (61/242, 95% CI 20%-31%) of patients had researched symptoms online themselves, not including patients whose family or friends searched on their behalf. Comparing our 95% confidence interval of 1.8% to 10.6% with Thomson et al’s 20% to 31%, the proportion found in our sample of lung cancer patients is clearly lower. This may be due to our participants being older with lower education levels than those in the Thomson et al study [17] because these factors have been related to lower levels of health-related Web use [20].

### Perceived Impact of Web Searches on the Pathway to Diagnosis

In our qualitative interview study, we explored how patients and their next-of-kin perceived the impact of prediagnosis Web searches on the events leading up to diagnosis. By mapping participants’ accounts onto the model developed by Walter et al [4], we showed that participants perceived an influence of the information they found online on all three intervals leading up to diagnosis (appraisal, help-seeking, diagnostic).

#### Appraisal Interval

In the appraisal interval, participants reportedly used online information to assess the seriousness of their symptoms and to identify possible causes. Previous research has suggested a rising importance of online health information on symptom appraisal processes; in the United States, for example, more than a third of adults report having used online information to identify the cause of symptoms [20].

Our findings suggest that participants searching in the appraisal interval had differing experiences with Web searches, with some reporting that the information led them to believe their symptoms were serious, whereas others reported that the information reassured them that symptoms were not serious.

Our interview findings tentatively suggest that search strategies may play an important role in how online information affects appraisal of symptoms. The participant who reportedly felt reassured had conducted a hypothesis-driven search strategy by researching symptoms paired together with her hypothesized cause “stopping smoking.” Therefore, search results were biased toward the hypothesized cause. Previous research [33] has suggested that searchers who use hypothesis-driven searches are prone to certain forms of bias, such as confirmation bias (starting with a hypothesis and confirming it) and premature termination bias (stopping after viewing only one topic). Further research on the differential effects of symptom-driven and hypothesis-driven searches is necessary to determine how search functions on health websites should be designed to enhance patients’ ability to appropriately appraise symptoms.

#### Help-Seeking Interval

When the Web was used in the help-seeking interval, participants reported that online information was used to inform their decision of whether to present to health services, and several participants reported that it encouraged them to make an appointment with a health care professional. Previous research confirms that most “online diagnosers” subsequently seek a professional medical opinion [20,34]. In Thomson et al’s [17] study with colorectal cancer patients, approximately a quarter of patients reportedly felt persuaded by the information found online to see a health professional. Furthermore, analyses of search engine log data have indicated that those who research symptoms online often subsequently show health care utilization intent (eg, by searching for clinics near their geographical area) [35].

Overall, previous research coupled with our results suggests that there may be a causal relationship between Web use and deciding to seeking help; this should be examined quantitatively in future research. Research in this area would be especially crucial for conditions such as lung cancer, in which earlier presentation to health services can maximize chances of survival.

#### Diagnostic Interval

Our analyses revealed two key findings regarding the diagnostic interval. Firstly, our results suggest that a division of this interval into two subintervals may be useful when examining the role of Web-based information. The first subinterval is the period from first consultation to the initiation of relevant diagnostic tests. The second subinterval begins with relevant diagnostic procedures and concludes with the final diagnosis. Our findings suggest the Web plays different roles in these two intervals.

When we examined searches that took place in the first sublevel, our findings suggest Web-based health information can empower patients and their families to appraise and challenge doctors’ advice and request further diagnostic procedures. This is particularly interesting because efforts to reduce patient delays to diagnosis in lung cancer have focused on encouraging presentation to health services [36-38] (ie, the appraisal and help-seeking interval). Little attention has been paid to the role patients play in the diagnostic interval.

Recent years have seen a shift toward patient-driven health care, with patients increasingly interested in decision making [39]. Web-based health information has been associated with this shift by increasing patients’ awareness of health professionals’ fallibility and uncertainties in diagnoses [40]. Thus, with the help of Web-based health information, patients may play an increasingly important role during the diagnostic interval. This is particularly important because our results indicate that individuals turn to the Web when they are dissatisfied with advice received from health care professionals and when they experience delays in obtaining a diagnosis. If future research and interventions focus on how the Web can be leveraged to support patients in this role, delays to diagnosis may be reduced.

In the second sublevel, after diagnostic tests were initiated, the Web was used to facilitate understanding of medical terms. The use of medical jargon in consultations, dissatisfaction with doctors’ communication skills, and the prevalence of low health literacy is well documented [41,42]. The majority of cancer patients prefer to be informed about their diagnosis [43]. Thus, the Web may prove a useful information resource before the diagnosis because it can facilitate understanding of medical jargon.

### Barriers to Prediagnosis Web Searches: Current and Future Importance of the Web Prior to a Lung Cancer Diagnosis

We explored barriers to using the Web for health information prior to diagnosis. This can help to assess the extent of the role the Web can play in lung cancer patients’ pathways to diagnosis and whether this role is likely to change in future.

The majority (79.6%, 90/113) of our sample reported not accessing the Web prior to diagnosis for a range of reasons. Although some of these barriers, such as perceived triviality of symptoms, may persist in the future, others are likely to change. For example, although some participants in our study reportedly preferred to defer to the doctor and not know details regarding their own health, engagement in health care is increasing and patients generally desire more detailed information from health professionals than they receive [43,44]. Furthermore, participants reported unfamiliarity with, or disinterest in, technology. This is unsurprising because the current cohort of those aged 65 and older, who are at highest risk for lung cancer, are less likely than any other age group to access the Internet [45]. Future lung cancer patients will be more familiar with the Web [46,47].

### Recommendations and Future Research

The association between prediagnosis Web searches and length of intervals in the pathway to diagnosis should be assessed statistically with a larger sample size. However, we will first need a validated, reliable measure of time intervals leading up to diagnosis. To date, no validated measure exists, and measures used in previous research have considerable limitations [48].

Furthermore, strategies to leverage the Web to encourage early presentation to health services should be investigated in future research. For example, our analyses indicate that individuals turn to the Web when they experience difficulty communicating with health professionals. Future research should therefore explore how health websites can provide information that will help facilitate patients’ communications with health professionals.

Our results suggest hypothesis-driven searches (search terms based on hypothesized conditions) and evidence-driven searches (search terms based on symptoms only) may have differing effects on how individuals evaluate symptoms. Future research should systematically investigate differential effects of hypothesis-driven and evidence-driven search strategies on symptom appraisal and subsequent help-seeking behavior. This will help to inform the development of Web-based symptom appraisal tools and search engine algorithms.

### Limitations

Our findings relied on retrospective, self-reported measures of patients, pertaining to events that took place prior to diagnosis. Cancer patients’ reports of the events leading up to diagnosis can be inconsistent [49]. Patients in our study had been diagnosed up to 6 months prior to study entry, and were asked to recall events before the diagnosis. Therefore, some of our measures may be subject to recall bias. For example, patients may not have been able to recall all search terms used during their prediagnosis Web searches. It was not feasible, however, to identify and recruit individuals with lung cancer prior to diagnosis.

Furthermore, patients may have been unaware of Web searches conducted by family/friends so this variable may be underreported in the survey because the survey was completed by patients. This could be addressed in future research by conducting a large-scale survey among patients and their next-of-kin.

Finally, as with all qualitative research, one must be cautious in generalizing from our purposive interview sample to the wider population of lung cancer patients.

### Conclusions

Because only 20.4% of the sample reported prediagnosis Web searches, it seems that the role of the Web prior to a diagnosis of lung cancer is still limited at present, but this proportion is likely to increase in the future, when barriers such as unfamiliarity with technology and unwillingness to be informed about one’s own health are likely to decrease.

Participants perceived an impact of their Web searches on symptom appraisal, the decision to present to health services, and on how they communicated with doctors and requested referrals to specialist care. This suggests using the Web prior to diagnosis may impact the appraisal, help-seeking, and diagnostic intervals referred to in Walter et al’s model [4], and thus on the length of time until a diagnosis is made. Although a quantitative analysis will be required to assess the statistical association between Web usage prediagnosis and the length of time from symptom occurrence to diagnosis, our study highlights potential mechanisms of how Web-based health information may influence pathways to diagnosis and can thus help to inform design of future research. The Web as a health information source is here to stay and, if it is to be an effective tool for health care systems, websites should use evidence-based designs to help potential patients make appropriate decisions about seeking medical treatment.

## Appendix

Multimedia Appendix 1 Excerpt of a framework matrix.
