# Supplementary material for: The Role of Web-Based Health Information in Help-Seeking Behavior Prior to a Diagnosis of Lung Cancer: A Mixed-Methods Study
Source: J Med Internet Res. 2017 Jun 8;19(6):e189. doi: 10.2196/jmir.6336 (PMC5481662; doi:10.2196/jmir.6336)
Supplement: Multimedia Appendix 1 [file jmir_v19i6e189_app1.pdf]

8. Web use for symptom apprai

	A : Demographics	B : 8.1. Perceived consequen ces of web search	C : 8.2. Reasons for or against Web use	D : 8.3. Search strategies	E : 8.4. What prompted the online resear ch
1 : 01_082_2<br>Web use = Web was used	Daughter of female patient, 84 years, widowed, secondary education, ex smoker, Web	Because of what she'd read she had a feeling it would be lung cancer. So none of what they were told came as a surprise. However she was shocked to hear the life	She uses the Internet because she is looking for anything she can do to help her mother. But it did 'scare her stiff'. She stopped going online when they started the treatment because now they have somebody to ask.	She typed "shoulder pain" into Google because she thought that's where the pain was. She searched every night for several days going through many websites, to find out if the symptoms (for LC) were all the same on different websites. [illegible]	She was prompted to use the internet because she was looking for something she could do to help her mother. She was initially prompted by the shoulder pain (started by typing symptoms) but also searched more after the [illegible]
2 : 01_101<br>Web use = Web was used	68 years, male, married, secondary school, retired, ex smoker, Web user	She used a process of elimination but this did not help her get to a conclusion, she did not feel like she had an idea what it could be after her research. Unclear how [illegible]	She feels like 'Google is the answer to everything'. Google is part of her life and she thinks it is fantastic. It's how she accesses any information. She feels like you have to utilise the resources that are there. Google replaces medical [illegible]	She (daughter) began by googling the symptom that was the 'main issue', the 'dry mouth'. She supplemented the information with info she got from friends who are health professionals. She also googled 'weak legs'. She used a [illegible]	
3 : 01_149_2<br>Web use = Web was used	Wife of patient (01_149), male, 62 years, retired, GCSE or equivalent, ex smoker, Web use	When they searched before seeing a doctor, she (wife) and the patient concluded after their online research that his symptoms were related to his smoking cessation	She used the internet as a backup, to answer questions she forgot to ask and to make herself aware of potentially relevant questions.	Pre help-seeking: She looked up night sweats, being awake at night, cough, together with stopping smoking. She read several websites and checked for congruence. She viewed congruence as a sign of 'that's	Pre help-seeking: He stopped smoking just before the symptoms occurred so this prompted her to look for the symptoms in conjunction with stopping smoking. She is a librarian and therefore experienced and
[illegible]	81 years, male	His children looked up	He (pt.) preferred not to use the	His children searched based on	He (the patient) had an x-ray and
